# First-Week Analysis after the Turkey Earthquakes: Demographic and Clinical Outcomes of Victims

**DOI:** 10.1017/S1049023X23000493

**Published:** 2023-06

**Authors:** Hıdır Sarı, Mehmet Özel, Mehmet Fatih Akkoç, Abdullah Şen

**Affiliations:** 1. Assistant Prof., Dicle University Faculty of Medicine Department of Public Health, Diyarbakır, Turkey; 2. Department of Emergency Medicine, Diyarbakır Gazi Yasargil Training and Research Hospital, University of Health Sciences, Diyarbakır, Turkey; 3. Assistant Prof., Dicle University Faculty of Medicine Department of Plastic Reconstructive and Aesthetic Surgery, Diyarbakır, Turkey; 4. Assistant Prof., Dicle University Faculty of Medicine Department of Emergency Medicine, Diyarbakır, Turkey

**Keywords:** crush injury, disaster, earthquake, Turkey, victims

## Abstract

**Background:**

During a major earthquake, escape attempts or collapsed buildings can result in injury, disability, and even death for victims. The aim of this study is to examine the demographic characteristics, clinical outcomes, and injuries of victims admitted to the emergency department within the first week after an earthquake.

**Methods:**

This is a retrospective observational study conducted on earthquake victims who were admitted to the emergency services of a tertiary medical faculty and a training and research hospital in the city of Diyarbakir, located in the Southeastern Anatolia Region of Turkey, from February 6 through February 12, 2023.

**Results:**

Of the eligible 662 earthquake victims, the mean age was 10.66 (SD = 4.78 [min 0, max 17]) in children, 36.87 (SD = 4.78 [min 18, max 63]) in adults, and 72.85 (SD = 5.83 [min 65, max 84]) in the elderly. Women constituted 52.8% of the victims, 19.7% were children, and 8.0% were elderly. Sixty-one percent (61.0%) of earthquake victims were admitted to emergency services in the first three days following the disaster; 37.7% of all victims were transferred from other affected cities to Diyarbakır. In all, 80.2% of the victims were admitted as survivors to the emergency services (36.8% were rescued under rubble, 40.1% with injuries while attempting to escape the earthquake, and 3.3% with nontraumatic reasons) and 19.8% were deceased under rubble. The majority of the 131 deceased victims were women (52.7%), 20.6% were children, and 7.6% were elderly. An estimated 38.3% of victims were hospitalized (20.9% in the ward and 17.4% in the intensive care unit [ICU]). For all age groups that survived under the rubble, the extremities were most injured (53.6% for children, 53.1% for adults, and 55.5% for the elderly). Of adult survivors, 26.6% needed only fluid therapy, renal replacement treatment (hemodialysis) was required 20.7%, and 11.8% required amputation. Of children survivors under the rubble, renal replacement treatment (hemodialysis) was required for only four, seven required amputation, and 12 needed only fluid resuscitation for crush injury. Of elderly survivors, two needed only fluid therapy, renal replacement treatment (hemodialysis) was required for two, and no amputation was required. Six patients survived under the rubble and died in the ICU.

**Conclusion:**

The definition of the demographic characteristics and clinical outcomes of earthquake patients is critical to the development of preparedness, response, and recovery policies for future disasters.

## Introduction

**Figure 1.**
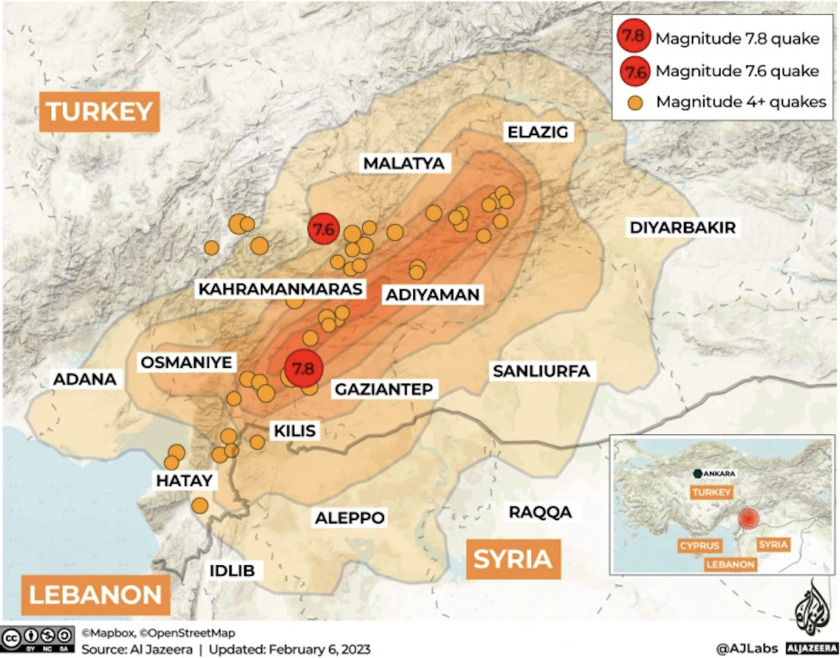
Areas Affected by the Earthquakes in Turkey.

On February 6, 2023, Turkey was struck by two of the most catastrophic earthquakes of the last century. During the early hours of February 6, Kahramanmaraş City in Turkey experienced its first earthquake with a magnitude of 7.7 on the Richter scale. A second earthquake with a magnitude of 7.6 struck the same region. In both earthquakes, the origin was approximately seven kilometers deep. More than 16 million people were affected by the earthquakes across 10 provinces, including Adana, Adıyaman, Diyarbakır, Gaziantep, Hatay, Kahramanmaraş, Kilis Osmaniye, and Şanlıurfa (Figure 1). Turkey suffered extensive damage from these earthquakes, which were among the most devastating disasters in several centuries. A Level 3 emergency has been declared by the Director-General of the World Health Organization (WHO; Geneva, Switzerland), Hans Kluge, after the earthquakes.^1^

The Turkish Ministry of Interior Disaster and Emergency Management Presidency (AFAD; Ankara, Turkey) released an official announcement informing the public that 2,724 aftershocks occurred in the first week following the earthquake, 31,643 people died, and 158,165 people were evacuated from the region.^2^ The AFAD announced on March 1, 2023 that more than 41 thousand lives had been lost and more than 115 thousand people had been injured.^3^ According to the Ministry of Environment, Urbanization, and Climate Change (Ankara, Turkey), over 1.5 million buildings were inspected during the damage assessment studies in the provinces affected by the earthquakes, and 202 thousand were determined to be demolished or heavily damaged in the emergency.^4^

There were several issues of concern about access to essential health services declared by the WHO in earthquake-affected regions of Turkey. Among the priority concerns reported were accessing the most vulnerable and affected populations in earthquake-affected areas, providing emergency trauma treatment and post-traumatic rehabilitation for the injured, and essential medicines, emergency kits, and supplies to meet urgent needs (particularly among women, children, elderly, and patients with chronic diseases).^5^

During the first earthquake in Turkey, people were asleep in their homes, and many were not able to get out, escape, or seek safety. As a result, thousands of buildings collapsed, trapping countless people under the rubble. According to a review, victims under the rubble could be extricated within hours to days of the initial injury. Due to the rush to provide emergency rescue and care, victims were separated, some of whom were taken to the nearest health facility. Additionally, earthquakes damaged hospitals in the disaster area, forcing victims to evacuate to other cities’ hospitals.^6^ The Health Ministry (Ankara, Turkey) reported 51,152 patients and injured people were transferred to other hospitals in the first days after the earthquakes.^7^

Despite disasters having similar magnitudes, vulnerabilities vary by community, so vulnerability research focuses on the factors that may cause damage to a community. Communities can assess their earthquake vulnerability based on the identified vulnerable characteristics. By doing so, the emergency response system will be strengthened, as well as the strategy for mitigating disaster losses.^8^ The literature lacks information about emergency health services, demographic characteristics, and clinical outcomes among disaster victims.^9,10^ The aim of this study is to examine the demographic characteristics, clinical outcomes, and injuries of victims admitted to the emergency department within the first week after an earthquake. Research findings could contribute to developing preparedness, response, and recovery policies for future disasters after an earthquake.

## Methods

### Overview

This is a retrospective observational study. The study involved patients affected by the earthquake who were admitted to the emergency services of a tertiary medical faculty and a training and research hospital in the city of Diyarbakir, located in the Southeastern Anatolia Region of Turkey, from February 6 through February 12, 2023 (the first week after the earthquakes). Patients with incomplete, inaccurate, or unreachable hospital records and emergency service admissions not related to the earthquakes were excluded. In accordance with the Helsinki Declaration, the Non-Interventional Clinical Research Ethics Committee of Dicle University Faculty of Medicine ethics committee approval (Diyarbakır, Turkey; Date: February 28, 2023 and Number: 55) and institutional permission were obtained before the study.

### Setting

In the Southeastern Region of Turkey, Diyarbakir province consists of 17 districts, four of which are centers. Based on the data provided by Turkish Statistical Institute (TUIK; Ankara, Turkey) for the year 2022, the province has 1,804,880 residents.^11^ A 546-bed training and research hospital and a 1,226-bed tertiary medical faculty are located in the city of Diyarbakir. Health services are provided by these hospitals in nine provinces throughout the southeast. These hospitals also serve as Level 1 trauma centers.

### Data Collection and Statistical Method

Each hospital’s emergency department and hospital electronic medical records were used to collect patient data. The data obtained from the study were analyzed using IBM SPSS Statistics for Windows, version 26.0 (IBM Corp.; Armonk, New York USA). Descriptive statistics were presented as percentages (%) and numbers (n); continuous variables were expressed as mean (standard deviation/SD [minimum(min)-maximum(max)]).

### Variables and Definitions

This study collected data on age, gender, the city affected by the earthquakes, the day of admission, the diagnosis, the emergency department’s clinical outcome, the type of injury (eg, escaping attempt from the earthquake, being trapped under rubble, or non-traumatic reason), the anatomical region of the injury, and the number of injuries. The treatment of the survivors under the rubble was documented, as well as their causes of death during hospitalization. Earthquake victims were defined as deceased (under rubble) and survivors. Survivors were divided into three categories: children, adults, and elderly.

## Results

A total of 662 earthquake victims had an average age of 34.60 (SD = 18.91 [min 0, max 84]) years, 36.31 (SD = 19.43) years in females, and 32.69 (SD = 18.15) years in males. The mean age was 10.66 (SD = 4.78 [min 0, max 17]) years in children, 36.87 (SD = 4.78 [min 18, max 63]) years in adults, and 72.85 (SD = 5.83 [min 65, max 84]) years in the elderly.

Women constituted 52.8% of the victims, 19.7% were children, and 8.0% were elderly. Sixty-one percent (61.0%) of earthquake victims were admitted to emergency services in the first three days following the disaster; 37.7% of all victims were transferred from other affected cities to Diyarbakır. Most transfers were from Adıyaman province (26.8% of all victims). In all, 80.2% of the victims were admitted as survivors to the emergency services (36.8% were rescued under rubble, 40.1% with injuries while attempting to escape the earthquake, and 3.3% nontraumatic reasons) and 19.8% were deceased under the rubble. Among the survivors under the rubble, 54.7% were women, 25.1% were children, and 5.3% were elderly. The majority of the 131 deceased victims were women (52.7%), 20.6% were children, and 7.6% were elderly. An estimated 38.3% of victims were hospitalized (20.9% in the ward and 17.4% in the intensive care unit [ICU]); Table 1.

**Table 1.** Clinical Characteristics and Outcomes of Earthquake Victims

	**Survivor** **(n = 530)**			**Deceased** **(n = 131)**	**Total** **n(%)**
	**Under Rubble** **n(%)**	**Escape Attempt** **n(%)**	**Non-Traumatic** **n(%)**	**Death Under Rubble** **n(%)**	
**Age Group**					
Children (<18 years)	61 (25.1)	33 (12.5)	9 (40.9)	27 (20.6)	130 (19.7)
Adult (18-64 years)	169 (69.6)	208 (78.4)	7 (31.8)	94 (71.8)	478 (72.3)
Elderly (≥65 years)	13 (5.3)	24 (9.1)	6 (27.3)	10 (7.6)	53 (8.0)
**Gender**					
Female	133 (54.7)	137 (51.7)	10 (45.5)	69 (52.7)	349 (52.8)
Male	110 (45.3)	128 (48.3)	12 (54.5)	62 (47.3)	312 (47.2)
**City of Earthquake**					
Adana	1 (0.4)	0 (0.0)	0 (0.0)	0 (0.0)	1 (0.2)
Adıyaman	121 (49.8)	37 (14.0)	17 (77.4)	2 (1.5)^c^	177 (26.8)
Diyarbakır	102 (42.0)	181 (68.3)	3 (13.6)	126 (96.2)	412 (62.3)
Gaziantep	0 (0.0)	2 (0.8)	1 (4.5)	0 (0.0)	3 (0.5)
Hatay	6 (2.5)	28 (10.6)	0 (0.0)	2 (1.5)^c^	36 (5.4)
Kahramanmaraş	9 (3.7)	12 (4.5)	1 (4.5)	1 (0.8)^c^	23 (3.5)
Malatya	3 (1.2)	5 (1.9)	0 (0.0)	0 (0.0)	8 (1.2)
Şanlıurfa	1 (0.4)	0 (0.0)	0 (0.0)	0 (0.0)	1 (0.2)
Kilis and Osmaniye	0 (0.0)	0 (0.0)	0 (0.0)	0 (0.0)	0 (0.0)
**Admission Day**					
1	86 (35.4)	126 (47.4)	1 (4.5)	23 (17.6)	236 (35.7)
2	44 (18.1)	40 (15.1)	0 (0.0)	15 (11.5)	99 (15.0)
3	25 (10.3)	28 (10.6)	4 (18.2)	11 (8.4)	68 (10.3)
4	36 (14.8)	15 (5.7)	3 (13.6)	5 (3.8)	59 (8.9)
5	21 (8.6)	18 (6.8)	2 (9.1)	5 (3.8)	46 (7.0)
6	15 (6.2)	28 (10.6)	1 (4.5)	11 (8.4)	55 (8.3)
7	16 (6.6)	10 (3.8)	11 (50.1)	61 (46.5)	98 (14.8)
**Clinical Outcome**					
Ward	85 (35.0)	47 (17.7)	6 (27.3)	0 (0.0)	138 (20.9)
ICU ^a^	92 (37.8)	10 (3.8)	13 (59.1)	0 (0.0)	115 (17.4)
Discharged from ED	66 (27.2)	208 (78.5)	3 (13.6)	0 (0.0)	277 (41.9)
Death Under Rubble	0 (0.0)	0 (0.0)	0 (0.0)	131 (100.0)	131 (19.8)
**Total** ^**b**^	**243 (36.8)**	**265 (40.1)**	**22 (3.3)**	**131 (19.8)**	**661 (100.0)**

Abbreviations: ED, emergency department; ICU, intensive care unit.

a Intubated (27), exitus (6).

b Percent (%) row.

c Deceased transferred to hospital family request.

Twenty-eight percent (28.0%) of survivors (n = 508) who were trapped under the rubble or attempting to escape the earthquake had two or more anatomical site injuries. The anatomical site of injury was 66.3% in the extremities and 14.8% in the head. For all age groups that survived under the rubble, the extremities were most injured (53.6% for children, 53.1% for adults, and 55.5% for the elderly). Of the survivors who were trapped under rubble and attempted to escape, 46.1% were hospitalized (20.1% ICU and 26.0% ward). Under the rubble, 57.3% of children and 38.5% of elderly survivors were admitted to intensive care. One child and the three elderly survivors who attempted to escape the earthquake were admitted to the ICU. Under the rubble, 52 adults were admitted to the ICU, six of whom died (Table 2).

**Table 2.** Injuries and Clinical Outcomes of Earthquake-Related Casualties

	Under Rubble (n = 243)			Escape Attempt (n = 265)			**Total** **(n = 508)**
	**Children** **(n = 61)** **<18 Years**	**Adult** **(n = 169)** **18-64 Years**	**Elderly** **(n = 13)** **≥65 Years**	**Children (n = 33)** **<18 Years**	**Adult** **(n = 208)** **18-64 Years**	**Elderly** **(n = 24)** **≥65 Years**	
	**n(%)**	**n(%)**	**n(%)**	**n(%)**	**n(%)**	**n(%)**	**n(%)**
**Number of Site Injury**							
1	30 (49.2)	82 (48.5)	8 (61.5)	31 (93.9)	193 (92.8)	22 (91.7)	366 (72.0)
2	18 (29.5)	55 (32.5)	5 (38.5)	2 (6.1)	15 (7.2)	2 (8.3)	97 (19.1)
3	13 (21.3)	29 (17.2)	0 (0.0)	0 (0.0)	0 (0.0)	0 (0.0)	42 (8.3)
4	0 (0.0)	3 (1.8)	0 (0.0)	0 (0.0)	0 (0.0)	0 (0.0)	3 (0.6)
**Site of Injury**							
Head	21 (20.0)	35 (12.1)	1 (5.6)	4 (11.4)	8 (3.6)	1 (3.8)	70 (10.0)
Trunk	22 (21.0)	65 (22.4)	6 (33.3)	1 (2.9)	7 (3.1)	2 (7.7)	103 (14.8)
Spinal	6 (5.7)	36 (12.4)	1 (5.6)	1 (2.9)	16 (7.2)	2 (7.7)	62 (8.9)
Extremity	56 (53.6)	154 (53.1)	10 (55.5)	29 (82.8)	192 (86.1)	21 (80.8)	462 (66.3)
**Clinical Outcome**							
Ward	14 (23.0)	66 (39.0)	5 (38.5)	6 (18.2)	37 (17.8)	4 (16.7)	132 (26.0)
ICU	35 (57.3)	52 (30.8)^a^	5 (38.5)	1 (3.0)	6 (2.9)	3 (12.5)	102 (20.1)
Discharged from ED	12 (19.7)	51 (30.2)	3 (23.0)	26 (78.8)	165 (79.3)	17 (70.8)	274 (53.9)

Abbreviations: ED, emergency department; ICU, intensive care unit.

a Exitus (6).

Due to crush syndrome, all survivors received fluid resuscitation. Of adult survivors, 26.6% needed only fluid therapy, renal replacement treatment (hemodialysis) was required for 20.7%, and 11.8% required amputation. Of children survivors under the rubble, renal replacement treatment (hemodialysis) was required for only four, seven required amputation, and 12 needed only fluid resuscitation for crush injury. Of elderly survivors, two needed only fluid therapy, renal replacement treatment (hemodialysis) was required for two, and no amputation was required (Table 3). Six patients survived under the rubble and died in the ICU. According to Table 4, the diagnosis of injury, the duration of hospitalization, and the causes of death were described.

**Table 3.** Treatment of Survivors Under the Rubble

	**Children** **(n = 61)** **<18 Years Old**	**Adult** **(n = 169)** **18-64 Years Old**	**Elderly** **(n = 13)** **≥65 Years Old**	**Total** **(n = 243)**
Only Fluid Resuscitation	12 (19.7%)	45 (26.6%)	2 (15.4%)	59 (24.3%)
Debridement	31 (50.8%)	69 (40.8%)	4 (30.8%)	104 (42.8%)
Fasciotomy	23 (37.7%)	39 (23.1%)	1 (7.7%)	63 (25.9%)
Amputation	7 (11.5%)	20 (11.8%)	0 (0.0%)	27 (11.1%)
Hemodialysis	4 (6.6%)	35 (20.7%)	2 (15.4%)	41 (16.9%)
Hyperbaric Oxygen Treatment	24 (39.3%)	27 (16.0%)	1 (7.7%)	52 (21.4%)
Other Surgery Treatment ^a^	20 (32.8%)	67 (39.6%)	8 (61.5%)	95 (39.1%)

a Surgical treatments for other conditions such as fracture treatment, tube thoracostomy, etc.

**Table 4.** Under the Rubble Survivors’ Hospitalization Length, Diagnosis, and Cause of Death in the ICU

Number	Gender	Age (year)	Diagnosis of Injury	Duration of Hospitalization (day)	Cause of Death
1	Female	45	Hypothermia, crush syndrome, crush injuries of lower limbs, abdominal injury	1	Acute Respiratory Failure
2	Female	58	Crush syndrome, crush injuries of lower limb	3	Crush Syndrome and Acute Renal Failure
3	Female	62	Crush syndrome, crush injuries of upper and lower limbs, traumatic brain injury, vertebral fracture	4	Crush Syndrome and Acute Renal Failure
4	Male	28	Crush syndrome, crush injuries of lower limb, traumatic brain injury, hemothorax	1	Multitrauma and Multiorgan Failure
5	Male	35	Crush syndrome, crush injuries of upper and lower limbs, vertebral fracture	1	Crush Syndrome and Acute Renal Failure
6	Male	48	Crush syndrome, crush injuries of lower limb, traumatic brain injury	2	Severe Traumatic Brain Injury

Abbreviation: ICU, intensive care unit.

## Discussion

In earthquakes, strong ground motions occur over short periods of time and lead to catastrophic destruction. Several devastating effects of earthquakes can be observed in society, such as their social, physical, and economic impacts.^12^ Globally, more than 60 million people have suffered earthquake damage and approximately 0.4 million have been killed by earthquakes during the last 30 years.^13^ In recent years, the deadliest earthquake disaster occurred in Haiti. Two hundred twenty (220) thousand people died in this disaster, which affected 3.7 million people.^14^ An occasional disaster with a high impact, the Turkey earthquake of February 2023 was among the deadliest earthquakes on record. The AFAD reported on March 1, 2023 that over 41 thousand people were dead (31,643 people were killed in the first week) and more than 115 thousand people were injured in the Kahramanmaraş-centered earthquakes.^2,3^

During an earthquake, unfortunate deaths were associated with very young and very old ages, poor socioeconomic status, and living indoors or in poorly constructed buildings. Most often, earthquake-related mortality and injuries were caused by building collapses, which led to soft tissue injuries, fractures, and crush injuries.^13^ An earthquake study in Yushu, China found 2,622 earthquake-related injuries among the victims. Of these, 54.81% were male with a median age of 36 years.^10^ An analysis of the Nepal Earthquake in 2015 found that 51.2% of the victims were women, and 17.2% were children. Due to being trapped under rubble, 48.9% of survivors suffered extremity crush injuries.^15^ In the present study, 80.2% of the 662 victims were admitted as survivors to the emergency services (36.8% had been rescued under rubble, 40.1% had suffered injuries while trying to escape the earthquake, and 3.3% for nontraumatic reasons) while 19.8% had died under the rubble. The majority of the 131 deceased victims were women (52.7%), 20.6% were children, and 7.6% were elderly.

Health care demand increases suddenly after earthquakes and facilities face the highest admissions within 72 hours of the earthquake.^10,15,16^ Sixty-eight percent (68.0%) of the 2,622 patients with earthquake-related injuries were admitted to the hospital within three days of the Yushu earthquake.^10^ During the Taiwan earthquake in 2016, 123 entrapped victims under collapsed buildings were extricated and transported to hospitals within 72 hours after the events.^17^ In the present study, approximately 61.0% of earthquake victims were admitted to emergency services in the first three days following the disaster.

In earthquake-prone areas, health facilities are particularly vulnerable because of direct and indirect damage (loss of utilities and infrastructure) that affects their ability to respond to emergencies.^13^ A Haitian earthquake in 2010 destroyed 60% of hospitals in the Ministry of Health, and more than 200 staff members perished. Most health care institutions in the earthquake area were unable to provide services.^18^ A district state hospital and two tertiary hospitals were destroyed in Hatay, Turkey after the earthquakes. There were also a number of private hospitals destroyed or permanently damaged in Hatay’s city center.^19^ A medical faculty hospital and three state hospitals were slightly damaged in Hatay. As a result of the recent earthquakes in Adana, moderate damage to the medical faculty hospital was found. Patients were transferred to other hospitals.^20^ One hundred fourteen (114) emergency response units and 25 field hospitals were established in the disaster area and 51,152 patients and injured were transferred to hospitals in other cities, according to the Ministry of Health.^8^ In the present study, 37.7% of all victims were transferred from other affected cities to Diyarbakır.

Most transfers were from Adıyaman province (26.8% of all victims). Adyaman facilities were only slightly damaged and usable after the earthquakes. With only one tertiary hospital in Adyaman, it experienced the highest number of admissions after the earthquake. As a result of this situation, it can be explained why most transfers were made from Adyaman to Diyarbakır.

Researchers have found that earthquake injuries are also influenced by the location where the victims were and the time when the earthquake occurred.^9,10,13^ A large number of people were asleep in their homes during the first earthquake in Turkey, and many of them were unable to escape or seek safety from the disaster. Numerous people were trapped under the rubble as a result of the collapse of thousands of buildings. Buried in rubble, victims can suffer crush injuries to their vital organs and extremities. In order to avoid complications, survivors from under the rubble needed a thorough evaluation, an early diagnosis, and aggressive treatment. The most common type of injury reported in the literature was crush extremity injuries that caused crush syndrome in people rescued alive from earthquake rubble.^9,10,13,17,21^ In this present study, the extremities were most injured in all age groups who survived under rubble (53.6% in children, 53.1% in adults, and 55.5% in the elderly).

The initial treatment for earthquake-related crush injuries included fluid-electrolyte balance, renal replacement therapy, and surgery interventions (ie, debridement, fasciotomy, or amputation).^9,17,21–23^ In the present study, due to crush injuries, all survivors received fluid resuscitation. Of adult survivors, 26.6% needed only fluid therapy, renal replacement treatment (hemodialysis) was required by 20.7%, and 11.8% required amputation. Of children survivors, renal replacement treatment (hemodialysis) was needed for only four, seven required amputations, and 12 were given only fluid resuscitation for crush injury. Of elderly survivors, two were given only fluid therapy, renal replacement therapy (hemodialysis) was required on two of them, and no amputation was necessitated. In the present study, existing crush injuries were managed and treated using a multidisciplinary approach (ie, fluid resuscitation, surgical intervention, or observation for possible rhabdomyolysis or acute kidney failure). Despite the multidisciplinary approach used, only six survivors under the rubble died during hospitalization in the first week following the disaster. As a result of this study, epidemiological knowledge of the demographics, injury types, treatment, and outcome will be provided, which will be very valuable in improving disaster relief.

## Limitation

The present study has several limitations. Based on the retrospective nature of this study, the fact that only victims with access to health care were included. Due to confusion and increased demand for health care during the time of the earthquake, there was a lack of record keeping or incomplete record keeping among the deceased. This caused in turn limited data information about the deceased’s demographics and causes of death. Achieving similar goals and objectives can be achieved through large-scale surveys that include data from earthquake victims in other affected provinces.

## Conclusion

A large number of people were injured, left homeless, or lost their lives in the Turkey earthquakes. Women, children, and the elderly were also affected. Most of those who died under the rubble were admitted to hospitals as deceased. To cope with the negative effects of the disaster, national and international support was required. Health facilities were also damaged by the earthquakes, creating unforeseen health needs. The vast majority of earthquake victims were admitted to emergency services in the first days following the disaster. In some survivors, crush injuries necessitated amputations and hemodialysis. It may be helpful to develop policies for preparedness, response, and recovery for future disasters, emergency response systems, and disaster loss reduction strategies if the demographic characteristics, clinical outcomes, and injuries of victims admitted to the emergency department within the first week are known. Also, demonstrating the social, psychological, physical, and economic effects of earthquake disasters may contribute to public health protection.

## References

[1] World Health Organization. Emergencies Türkiye and Syria Earthquakes. https://www.who.int/europe/emergencies/situations/turkiye-and-syria-earthquakes. Accessed March 1, 2023.

[2] Ministry of Interior Disaster and Emergency Management Presidency. Press bulletin 13.02.2023 about the earthquake in Kahramanmaraş - 29. https://en.afad.gov.tr/press-bulletin-29-about-the-earthquake-in-kahramanmaras. Accessed March 1, 2023.

[3] Ministry of Interior Disaster and Emergency Management Presidency. Press bulletin 01.03.2023 about the earthquake in Kahramanmaraş - 36. https://en.afad.gov.tr/press-bulletin-36-about-the-earthquake-in-kahramanmaras. Accessed March 2, 2023.

[4] Republic of Türkiye Ministry of Environment, Urbanization and Climate Change. Press bulletin 28.02.2023. https://www.csb.gov.tr/bakan-kurum-582-bin-bagimsiz-bolum-ve-202-bin-binanin-acil-yikilacak-agir-hasarli-veya-yikik-oldugu-tespitini-yaptik-bakanlik-faaliyetleri-3845. Accessed March 2, 2023.

[5] World Health Organization. Türkiye earthquake: external situation report no.1: 13–19 February 2023. https://www.who.int/europe/publications/i/item/WHO-EURO-2023-7145-46911-68441. Published 2023. Accessed March 1, 2023.

[6] Schultz CH , Koenig KL , Noji EK. A medical disaster response to reduce immediate mortality after an earthquake. N Engl J Med. 1996;334(7):438–444. 8552147 10.1056/NEJM199602153340706

[7] Republic of Türkiye Ministry of Health. Health Minister Dr. Koca Shared the latest situation of health services in earthquake regions. www.saglik.gov.tr/EN,94845/health-minister-dr-koca-shared-the-latest-situation-of-health-services-in-earthquake-regions.html. Accessed March 1, 2023.

[8] Jeong S , Yoon DK. Examining vulnerability factors to natural disasters with a spatial autoregressive model: the case of South Korea. Sustainability. 2018;10(5):1651.

[9] Bulut M , Fedakar R , Akkose S , et al. Medical experience of a university hospital in Turkey after the 1999 Marmara earthquake. Emerg Med J. 2005;22(7):494–498. 15983085 10.1136/emj.2004.016295 PMC1726859

[10] Kang P , Zhang L , Liang W , et al. Medical evacuation management and clinical characteristics of 3,255 inpatients after the 2010 Yushu earthquake in China. J Trauma Acute Care Surg. 2012;72(6):1626–1633. 22695432 10.1097/TA.0b013e3182479e07

[11] Turkish Statistical Institute. National population data. https://data.tuik.gov.tr/Bulten/Index?p=Adrese-Dayali-Nufus-Kayit-Sistemi-Sonuclari-2022-49685. Accessed March 3, 2023.

[12] Wu J , He X , Li Y , Shi P , Ye T , Li N. How earthquake-induced direct economic losses change with earthquake magnitude, asset value, residential building structural type and physical environment: an elasticity perspective. J Environ Manage. 2019;231:321–328. 30359897 10.1016/j.jenvman.2018.10.050

[13] Doocy S , Daniels A , Packer C , Dick A , Kirsch TD. The human impact of earthquakes: a historical review of events 1980-2009 and systematic literature review. PLoS Curr. 2013;5. 10.1371/currents.dis.67bd14fe457f1db0b5433a8ee20fb833 PMC3644288 23857161

[14] EM-DAT International Disaster Database. Haiti profile. 2023. www.emdat.be/emdat_atlas/sub_html_pages/sub_html_HTI.html. Accessed March 4, 2023.

[15] Moitinho de Almeida M. “Recovering, not recovered.” Hospital disaster resilience: a case-study from the 2015 earthquake in Nepal. Glob Health Action. 2022;15(1):2013597. 35138232 10.1080/16549716.2021.2013597 PMC8843347

[16] Macintyre AG , Barbera JA , Smith ER. Surviving collapsed structure entrapment after earthquakes: a “time-to-rescue” analysis. Prehosp Disaster Med. 2006;21(1):4–17. 16602260 10.1017/s1049023x00003253

[17] Pan ST , Cheng YY , Wu CL , et al. Association of injury pattern and entrapment location inside damaged buildings in the 2016 Taiwan earthquake. J Formos Med Assoc. 2019;118(1 Pt 2):311–323. 29857951 10.1016/j.jfma.2018.05.012

[18] Arnaouti MKC , Cahill G , Baird MD , et al; Haiti Disaster Response – Junior Research Collaborative (HDR-JRC). Medical disaster response: a critical analysis of the 2010 Haiti earthquake. Front Public Health. 2022;10:995595. 36388301 10.3389/fpubh.2022.995595 PMC9665839

[19] Turkish Enterprise and Business Confederation (TURKONFED)-2023 Kahramanmaraş Earthquake-Pre-Assessment and Status Report. https://turkonfed.org/tr/detay/3937/2023-kahramanmaras-depremi-afet-on-degerlendirme-durum-raporu. Published 2023. Accessed March 4, 2023.

[20] Republic of Türkiye Ministry of Health. Health Minister Dr. Fahrettin Koca made a press statement at the field hospital established in front of Hatay Training and Research Hospital. https://www.saglik.gov.tr/Genel/MansetHaberList.aspx. Accessed March 1, 2023.

[21] Gul A , Andsoy II. Performed surgical interventions after the 1999 Marmara Earthquake in Turkey and their importance regarding nursing practices. J Trauma Nurs. 2015;22(4):218–222. 26165875 10.1097/JTN.0000000000000136

[22] Dai ZY , Li Y , Lu MP , et al. Clinical profile of musculoskeletal injuries associated with the 2008 Wenchuan earthquake in China. Ulus Travma Acil Cerrahi Derg. 2010;16(6):503–507. 21153941

[23] Jiang J , Li Y , Huang X , et al. Lessons learnt from the Wenchuan earthquake: performance evaluation of treatment of critical injuries in hardest-hit areas. J Evid Based Med. 2012;5(3):114–123. 23672218 10.1111/j.1756-5391.2012.01186.x

